# Prognostic potential of neutrophil-to-lymphocyte ratio for appendicular skeletal muscle mass reduction in males aged 70 and older

**DOI:** 10.18632/aging.206217

**Published:** 2025-03-06

**Authors:** Ying-Jen Chen, Chieh-Li Yen, Chern-Horng Lee, Kuo-Chen Liao, Ji-Tseng Fang, Tz-Shiu Tsai, Yi-Ching Chen, Chun-Yen Lin

**Affiliations:** 1 Geriatric Medical Center, Linkou Medical Center, Chang Gung Memorial Hospital, Taoyuan, Taiwan; 2 Department of Geriatrics and General Internal Medicine, Linkou Medical Center, Chang Gung Memorial Hospital, Taoyuan, Taiwan; 3 Department of Nephrology, Linkou Medical Center, Chang Gung Memorial Hospital, Taoyuan, Taiwan; 4 Division of Hepatology, Department of Gastroenterology and Hepatology, Linkou Medical Center, Chang Gung Memorial Hospital, Taoyuan, Taiwan; 5 College of Medicine, Chang Gung University, Taoyuan, Taiwan

**Keywords:** sarcopenia, skeletal muscle, inflammatory index, neutrophil-to-lymphocyte ratio, NLR

## Abstract

Inflammation plays a pivotal role in the age-related decline of skeletal muscle mass, leading to sarcopenia in the elderly. The prevalence of sarcopenia notably increases among males aged ≥ 70. However, it remains unclear whether inflammatory indexes are associated with the reduction in skeletal muscle mass in the elderly population.

Thirty-one males aged ≥ 70, without severe diseases or dementia, were enrolled in the study. They underwent muscle mass measurements, physical measurements, and hematological tests at the onset of the study and after a one-year follow-up.

Twenty-eight participants were successfully followed for one year. Appendicular skeletal muscle mass index (ASMI) decreased by 3.30 ± 2.41% in 14 participants and increased by 2.66 ± 1.61% in the other 14 participants compared to baseline levels. The baseline neutrophil-to-lymphocyte ratio (NLR) was 2.14 ± 0.56 in the ASMI-decreased group and 1.66 ± 0.62 in the ASMI-increased group. A statistically significant negative correlation was found between baseline NLR and the change in ASMI in linear regression analyses. The area under the curve (AUC) of the baseline NLR for predicting ASMI decline was 0.724, with an optimal sensitivity of 64.3% and specificity of 78.6% at a cut-off value of 1.94.

NLR emerged as a potential prognostic marker for ASMI reduction in elderly males. However, further studies are necessary to assess its clinical utility.

## INTRODUCTION

Loss of skeletal muscle mass, strength, and/or function leads to sarcopenia, which has been associated with fall-related injuries [1], decreased quality of life [2], and poor outcomes in various diseases, including coronavirus disease 2019 (COVID-19) [3], coronary artery disease [4], non-small-cell lung cancer [5, 6], and renal cell carcinoma [7]. Cao and colleagues estimated the prevalence of sarcopenia to be 1.5% among men aged 60-69, increasing notably to 9.6% among men aged 70-79 and 33.1% among those aged 80 and older [8]. Therefore, monitoring skeletal muscle mass, strength, and function is essential for the elderly. However, current methods for assessing muscle mass, such as dual-energy x-ray absorptiometry (DXA) or bioelectrical impedance analysis (BIA) [9], are challenging to implement regularly on a large scale. Common screening tools for sarcopenia include the Strength, Assistance with walking, Rise from a chair, Climb stairs, and Falls (SARC-F) questionnaire and calf circumference (CC) measurement. These tools primarily focus on the physical outcomes of sarcopenia, and their effectiveness in screening for the loss of skeletal muscle mass remains unclear. Therefore, a more convenient approach for large-scale muscle mass assessment may be necessary.

Inflammation plays a crucial role in the reduction of skeletal muscle mass [10], contributing to sarcopenia in the elderly [11]. Inflammatory indexes such as the neutrophil-to-lymphocyte ratio (NLR), platelet-to-lymphocyte ratio (PLR), and systemic immune-inflammation index (SII) have been associated with sarcopenia risk [12–16]. Additionally, studies have shown that neutrophils, platelets, and lymphocytes are involved in skeletal muscle atrophy, regeneration, and repair [17–19]. These inflammatory indexes, derived from absolute counts of peripheral blood cells, suggest the potential for monitoring sarcopenia risk during routine hematological tests. However, the correlation between these inflammatory indexes and the loss of skeletal muscle mass remains unclear.

Reduced skeletal muscle mass is often underestimated among the elderly, as it is weakly linked to declined physical performance [20]. To intervene and prolong skeletal muscle health in the elderly as early as possible, this study aimed to identify markers for evaluating skeletal muscle mass reduction. Specifically, we aimed to elucidate the potential of inflammatory indexes for screening the elderly with reduced skeletal muscle mass for further examination.

## RESULTS

### Baseline characteristics of participants with increased and decreased ASMI

**Figure 1:**
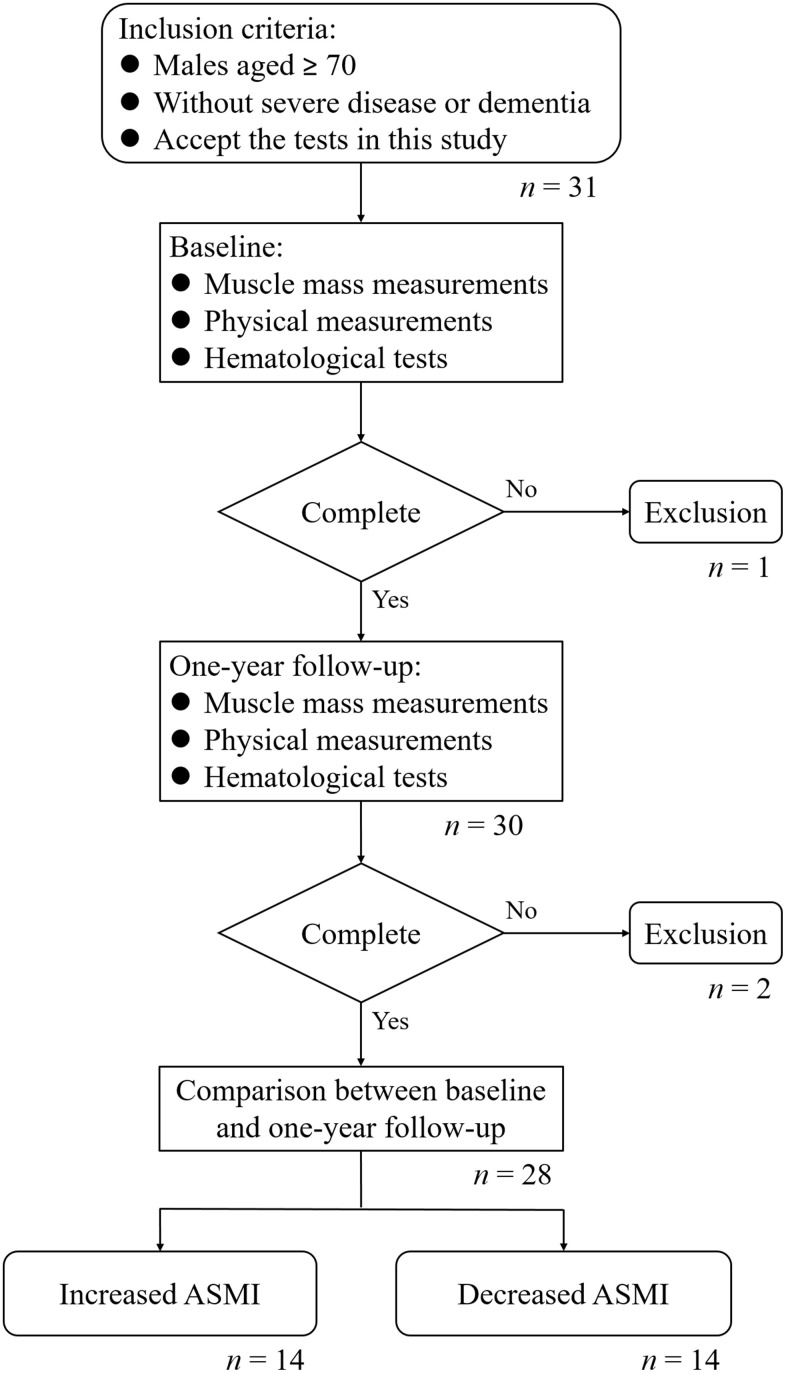
Flowchart of this study.

Out of the 31 participants enrolled in this study, 28 were successfully followed for one year and completed all tests (Figure 1). It was observed that ASMI increased by 2.66 ± 1.61% in 14 participants over the year, while it decreased by 3.30 ± 2.41% in the other 14 participants. The prevalence of sarcopenia was 57.14% among participants with increased ASMI and 28.57% among those with decreased ASMI. However, the prevalence of sarcopenia was not significantly associated with ASMI change (*p*-value = 0.127). To identify potential prognostic markers of ASMI reduction, the baseline physical measurements and hematological test results of participants with increased and decreased ASMI were retrospectively compared. Correlation coefficients between the baseline levels of each characteristic and ASMI change were analyzed (Table 1). Significantly negative correlations between ASMI change and BMI, CC, neutrophil proportion in WBC, and NLR were noted, and a positive correlation between ASMI change and lymphocyte proportion in WBC was observed. The characteristics mentioned above exhibited apparent differences between the baseline levels of participants with increased and decreased ASMI.

**Table 1:** Relationships between baseline characteristics and ASMI changes.

**Characteristics**	**Increased ASMI** **(*n* = 14)**	**Decreased ASMI** **(*n* = 14)**	***p*-value^a^**	**Correlation with ASMI change**	
				***r***	***p*-value**
Sarcopenia, *n* (%)			0.127		
Yes	8 (57.14)	4 (28.57)			
No	6 (42.86)	10 (71.43)			
ASMI change, %	2.66 ± 1.61	-3.30 ± 2.41			
Age, years	76.21 ± 2.46	76.43 ± 6.54	0.910	-0.036	0.857
Height, cm	164.73 ± 5.97	165.63 ± 6.79	0.713	-0.175	0.372
BMI, kg/m^2^	**25.18 ± 2.18**	**28.48 ± 4.73**	**0.029**	**-0.478**	**0.010**
CC, cm	**34.92 ± 2.12**	**37.15 ± 3.05**	**0.041**	**-0.493**	**0.010**
WBC, 10^9^/L	6.63 ± 2.12	6.36 ± 1.67	0.717	0.064	0.748
Neutrophil, 10^9^/L	3.68 ± 1.50	3.83 ± 1.02	0.760	-0.090	0.649
Neutrophil/WBC, %	**54.16 ± 9.02**	**60.24 ± 5.73**	**0.043**	**-0.413**	**0.029**
Monocyte, 10^9^/L	0.42 ± 0.16	0.44 ± 0.12	0.727	0.112	0.571
Monocyte/WBC, %	6.27 ± 1.31	7.02 ± 1.44	0.162	0.020	0.921
Lymphocyte, 10^9^/L	2.26 ± 0.62	1.90 ± 0.73	0.168	0.270	0.165
Lymphocyte/WBC, %	**35.54 ± 8.68**	**29.34 ± 5.34**	**0.031**	**0.380**	**0.046**
Platelet, 10^9^/L	217.21 ± 71.63	192.43 ± 48.81	0.295	0.308	0.111
NLR	**1.66 ± 0.62**	**2.14 ± 0.56**	**0.038**	**-0.428**	**0.023**
MLR	**0.18 ± 0.05**	**0.25 ± 0.06**	**0.007**	-0.279	0.150
PLR	101.48 ± 38.46	114.98 ± 56.83	1.000	-0.045	0.819
SII	348.07 ± 153.56	403.49 ± 128.54	0.310	-0.081	0.682
SIRI	0.71 ± 0.39	0.92 ± 0.31	0.121	-0.192	0.328
PIV	151.83 ± 104.86	178.29 ± 85.98	0.472	0.018	0.928

Characteristics, excluding sarcopenia prevalence, were presented as mean ± S.D. Statistical significance was denoted by bold values.

^a^Comparison between numbers or levels in study participants with increased and decreased ASMI.

### One-year follow-up of participants with increased and decreased ASMI

Changes in physical and hematological characteristics from baseline to one-year follow-up were analyzed in participants with increased and decreased ASMI, and their correlation coefficients with ASMI change were evaluated (Table 2). A significantly positive correlation was revealed between changes in BMI and ASMI, and a negative correlation was revealed between changes in systemic inflammation response index (SIRI) and ASMI. However, only the decline of BMI from baseline to one-year follow-up in participants with decreased ASMI was a significant change.

**Table 2:** Relationships between changes in characteristics from baseline to one-year follow-up and ASMI changes.

**Characteristics**	**ASMI^a^**	**One-year follow-up**	***p*-value^b^**	**Change from baseline**	**Correlation with ASMI change**	
					***r***	***p*-value**
BMI, kg/m^2^	+	25.29 ± 1.99	0.608	0.11 ± 0.76	**0.489**	**0.008**
	-	**27.91 ± 4.68**	**0.010**	-0.57 ± 0.71		
CC, cm	+	34.75 ± 1.94	0.786	-0.19 ± 1.79	0.319	0.112
	-	36.14 ± 2.60	0.228	-0.69 ± 1.96		
WBC, 10^9^/L	+	6.34 ± 2.07	0.194	-0.29 ± 0.80	-0.352	0.066
	-	6.69 ± 1.97	0.433	0.32 ± 1.49		
Neutrophil, 10^9^/L	+	3.53 ± 1.56	0.511	-0.15 ± 0.84	-0.342	0.075
	-	4.12 ± 1.66	0.460	0.29 ± 1.44		
Neutrophil/WBC, %	+	53.70 ± 12.00	0.828	-0.46 ± 7.83	-0.140	0.479
	-	60.81 ± 9.99	0.811	0.57 ± 8.72		
Monocyte, 10^9^/L	+	0.41 ± 0.17	0.631	-0.01 ± 0.08	-0.266	0.172
	-	0.46 ± 0.13	0.415	0.02 ± 0.10		
Monocyte/WBC, %	+	6.36 ± 1.13	0.742	0.09 ± 0.95	0.036	0.855
	-	6.91 ± 1.27	0.761	-0.11 ± 1.38		
Lymphocyte, 10^9^/L	+	2.14 ± 0.63	0.249	-0.13 ± 0.40	0.014	0.945
	-	1.86 ± 0.66	0.761	-0.04 ± 0.44		
Lymphocyte/WBC, %	+	35.87 ± 12.37	0.863	0.34 ± 7.14	0.128	0.515
	-	28.61 ± 9.01	0.743	-0.73 ± 8.12		
Platelet, 10^9^/L	+	204.93 ± 54.97	0.305	-12.29 ± 43.04	-0.112	0.572
	-	181.43 ± 33.31	0.213	-11.00 ± 31.42		
NLR	+	1.72 ± 0.73	0.675	0.06 ± 0.54	-0.357	0.062
	-	2.49 ± 1.36	0.300	0.35 ± 1.06		
MLR	+	0.19 ± 0.06	0.595	0.01 ± 0.05	-0.249	0.202
	-	0.26 ± 0.09	0.397	0.02 ± 0.07		
PLR	+	102.45 ± 37.17	0.886	0.97 ± 24.67	-0.046	0.815
	-	110.42 ± 51.34	0.826	-4.56 ± 30.35		
SII	+	340.83 ± 172.58	0.875	-7.24 ± 168.28	-0.283	0.144
	-	433.43 ± 186.85	0.568	29.94 ± 191.33		
SIRI	+	0.74 ± 0.51	0.778	0.03 ± 0.40	**-0.414**	**0.028**
	-	1.15 ± 0.75	0.241	0.23 ± 0.69		
PIV	+	152.64 ± 131.81	0.331	0.81 ± 118.57	-0.293	0.130
	-	199.79 ± 100.93	0.492	21.50 ± 113.76		

All characteristics were presented as mean ± S.D. Statistical significance was denoted by bold values.

^a^ASMI (+) or (-) indicated ASMI increase or decrease.

^b^Comparison between levels at baseline and one-year follow-up.

### Potential factor for predicting one-year ASMI reduction

The baseline levels of NLR, BMI and CC were subjected to univariable regression analyses for ASMI change, with all variables showing significantly negative association. These baseline variables were then included in multivariable regression analyses, where baseline NLR exhibited the strongest negative association with ASMI change among all baseline variables (Table 3).

**Table 3:** Univariable and multivariable regression analyses of ASMI change.

**Baseline variables**	**Univariable regression analysis**				**Multivariable regression analysis**			
	**B**	**95% CI**		***p*-value**	**B**	**95% CI**		***p*-value**
BMI, kg/m^2^	-0.436	-0.759	-0.113	0.010*	-0.326	-0.642	-0.009	0.044*
CC, cm	-0.646	-1.127	-0.166	0.010*	-0.385	-0.845	0.075	0.096
NLR	-2.474	-4.579	-0.368	0.023*	-2.106	-3.887	-0.325	0.023*

*p-value < 0.05

**Figure 2:**
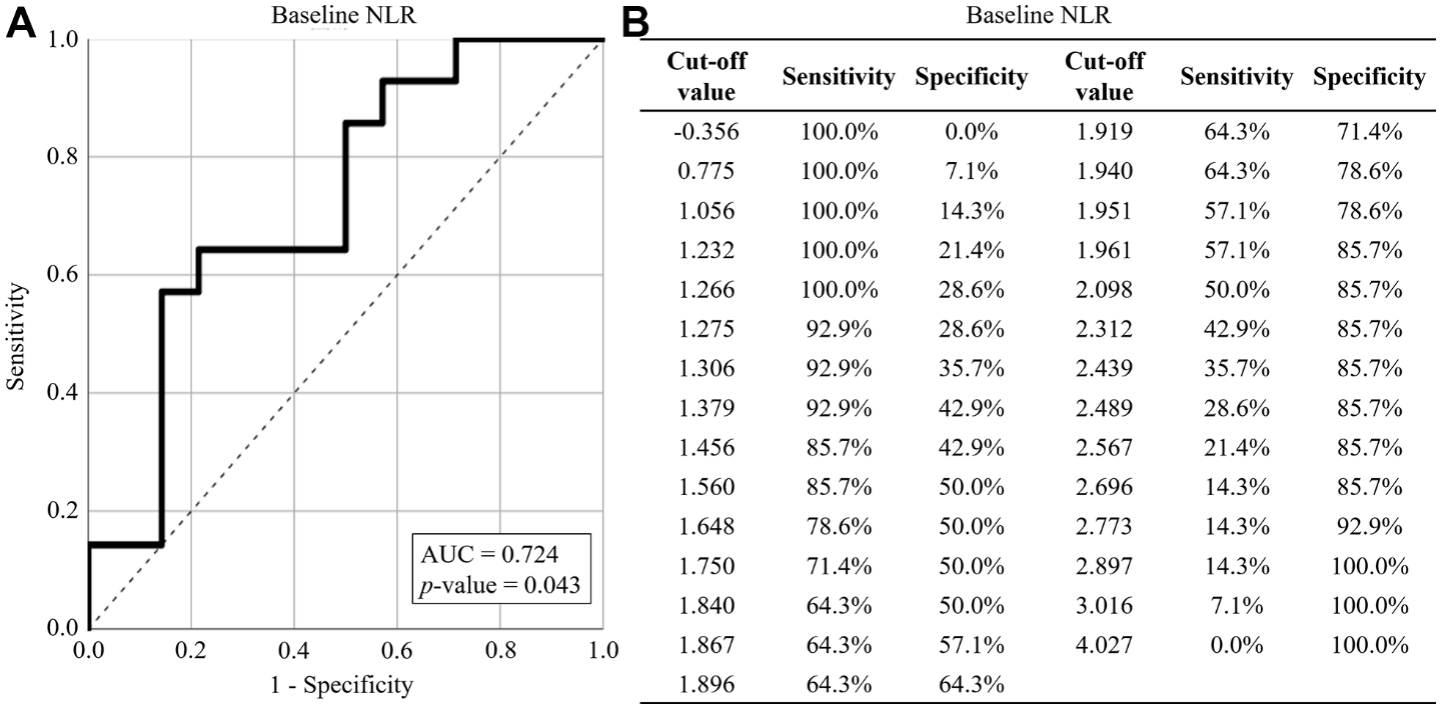
**ROC curve analysis for ASMI reduction.** (**A**) The performance of baseline NLR in predicting ASMI reduction. (**B**) The sensitivity and specificity of baseline NLR in predicting ASMI reduction. The baseline NLR was represented by solid lines, while reference lines were depicted by dashed lines. AUC: area under the curve.

The baseline NLR was further analyzed using ROC curve analyses to evaluate its relationship with ASMI change. The area under the curve (AUC) for baseline NLR was 0.724 (*p*-value = 0.043) (Figure 2A). The optimal sensitivity (64.3%) and specificity (78.6%) for predicting ASMI decline were observed when the cut-off was set at 1.94 (Figure 2B).

## DISCUSSION

Chronic systemic inflammation is a key aging-related phenotypes [21]. Aging-related inflammatory cytokines have been shown to contribute to the loss of skeletal muscle mass. For instance, tumor necrosis factor-α (TNF-α) activates the signaling pathway of muscle cell pyroptosis [22]. NLR, derived from the absolute counts of peripheral neutrophils and lymphocytes, is a systemic inflammatory index. The normal range of NLR is considered to be between 1.00 and 2.00, with values higher than 3.00 or lower than 0.70 deemed pathological [23]. In this study, we found that a higher NLR level in males aged ≥ 70, even within normal range, was associated with a ASMI decrease in the following year. In this study, the baseline NLR was 2.14 ± 0.56 in ASMI-decreased participants and 1.66 ± 0.62 in ASMI-increased participants. While WBC counts were similar between ASMI-decreased and ASMI-increased participants, the higher NLR level in ASMI-decreased participants resulted from a relatively higher neutrophil proportion and a lower lymphocyte proportion compared to those in ASMI-increased participants. Previously, Nakanishi and colleagues demonstrated that neutrophils lead to muscle atrophy in animal models [17], and Slaets and colleagues reported that T lymphocytes are crucial for muscle regeneration [24].

Yoon and colleagues investigated the relationship between skeletal muscle mass and inflammatory markers in Korean adults, finding that males with low muscle mass exhibited higher NLR compared to those with normal muscle mass in a cross-sectional study [25]. In our study, we highlighted a correlation between higher NLR and ASMI reduction after one year in Taiwanese males aged ≥ 70. The prognostic potential of NLR underscores its importance for early intervention to prevent further muscle loss among the elderly.

Based on ASMI, handgrip strength, and 4-metre gait speed measurements, we identified 8 (57.14%) sarcopenic participants among ASMI-increased participants and 4 (28.57%) sarcopenic participants among ASMI-decreased participants. However, no significant relationship between sarcopenia and ASMI reduction was observed. It is documented that exercise interventions, particularly resistance training and electromyostimulation, are beneficial for improving muscle mass and strength in sarcopenic patients [26, 27]. Previous studies have reported that high NLR is associated with sarcopenia [12, 13, 16]. NLR has been developed as a prognostic marker of sarcopenia, with the cut-off value varying depending on the study population. For example, Hu and colleagues reported that hospitalized renal cell carcinoma patients with NLR > 2.88 have a higher risk of sarcopenia [13], while Borges and colleagues found that hospitalized cancer patients with NLR ≥ 6.5 have a higher risk of sarcopenia [12]. In our study, which enrolled elderly patients without severe illness or dementia, we found that males aged ≥ 70 with NLR ≥ 1.94 had a higher risk of ASMI reduction. These findings suggest that sarcopenic patients should not give up on strengthening their muscles, as sarcopenia does not necessarily lead to further muscle mass decline. Additionally, the NLR of elderly men should be regularly evaluated, and changes in NLR should be closely monitored, even if they have not reached the threshold of high sarcopenia risk.

We observed that the baseline BMI and CC were 25.18 ± 2.18 and 34.92 ± 2.12 cm, respectively, in ASMI-increased participants, whereas these values were 28.48 ± 4.73 and 37.15 ± 3.05 cm in ASMI-decreased participants. Low BMI has been associated with a higher risk of probable sarcopenia [28], and AWGS identifies males with CC < 34 cm as being at high risk of sarcopenia [9]. However, the relationships between BMI and CC and muscle mass reduction have not been fully elucidated. The inverse relationships we observed between baseline BMI and CC and ASMI change suggest that males with higher BMI and CC tend to experience a decrease in ASMI after one year. An apparent decline in BMI, but not in CC, was then observed along with ASMI reduction. On one hand, this may be because males with higher ASMI require more or more intense exercise to increase or maintain their ASMI. On the other hand, the mean BMI in this study were relatively higher, and a large proportion of participants met the criteria for obesity. The negative correlation between baseline BMI and subsequent ASMI decline suggests that obesity may play a role in the progression of sarcopenia, commonly referred to as sarcopenic obesity. Therefore, personalized exercise recommendations for the elderly, considering their BMI and CC, are important.

There are limitations to this study. First, it remains unclear whether the higher NLR is a cause or a consequence of ASMI decrease. Skeletal muscle has been reported to secrete myokines that attenuate inflammation [29], but it is unknown whether the proportions of neutrophils and lymphocytes in WBC are directly or indirectly affected by skeletal muscle. Continuous monitoring of NLR and ASMI changes in the study participants over several years is necessary to clarify their relationship. Second, our limited sample size may have introduced bias into the results. Further investigations with a larger cohort are needed to validate our findings.

## CONCLUSION

The communication of skeletal muscles and other organs has been increasingly recognized over the past few decades. Skeletal muscle is not only essential for body mobility but also plays a crucial role in immunity [24], glucose metabolism [30], bone metabolism [31], cognitive function [32], and tumor suppression [33]. These issues are major medical concerns for the elderly, making the monitoring of skeletal muscle mass important for this population. A reliable prognostic marker of skeletal muscle mass reduction could assist geriatricians in early medical intervention. In this study, we identified NLR as a potential prognostic marker for ASMI decline, with current data suggesting that a higher NLR (>1.94) might predict muscle mass reduction over the following year. However, due to the limited sample size of this study, the clinical utility of NLR requires further investigation in larger cohorts.

## MATERIALS AND METHODS

### Study participants

Given the strikingly elevated prevalence of sarcopenia in males aged ≥ 70 as reported [8], males aged ≥ 70 without severe diseases or dementia were enrolled in this study. They underwent muscle mass measurements, physical measurements, and hematological tests. Twenty-one participants were recruited from the Department of Geriatrics and General Internal Medicine, Linkou Medical Center, Chang Gung Memorial Hospital, and 10 participants were recruited through medical examinations in the community. The participants were followed for one year, and those unable to complete the muscle mass measurements, physical measurements, or hematological tests were excluded from the study. No participant experienced a severe infection during the study.

### Sarcopenia definition

According to the consensus of the Asian Working Group for Sarcopenia (AWGS) in 2019 [9], participants in this study were classified as sarcopenic if their appendicular skeletal muscle mass index (ASMI), which was calculated as appendicular skeletal muscle mass divided by the square of height, was lower than 7 kg/m^2^, accompanied by handgrip strength lower than 28 kg or a 4-metre gait speed slower than 1 m/s.

### Data collection

Clinical data, including age, height, body mass index (BMI), and CC, were collected when participants visited the geriatric outpatient clinic or underwent medical examinations. The ASMI of participants recruited from Chang Gung Memorial Hospital was measured by DXA, while the ASMI of participants recruited from medical examinations in the community was measured by BIA. Peripheral blood samples were collected by trained professionals to analyze white blood cell (WBC) and platelet counts. Inflammatory indexes were calculated as follows: NLR = neutrophil count (10^9^/L)/lymphocyte count (10^9^/L); monocyte-to-lymphocyte ratio (MLR) = monocyte count (10^9^/L)/ lymphocyte count (10^9^/L); PLR = platelet count (10^9^/L)/lymphocyte count (10^9^/L); SII = neutrophil count (10^9^/L) × platelet count (10^9^/L)/lymphocyte count (10^9^/L); SIRI = neutrophil count (10^9^/L) × monocyte count (10^9^/L)/lymphocyte count (10^9^/L); pan-immune-inflammation value (PIV) = neutrophil count (10^9^/L) × monocyte count (10^9^/L) × platelet count (10^9^/L)/lymphocyte count (10^9^/L).

### Statistical analysis

The prevalence of sarcopenia among study participants was analyzed using the Chi-squared test to evaluate the relationship between sarcopenia and ASMI change. Quantitative variables were expressed as mean ± standard deviation and analyzed using the Kolmogorov-Smirnov test to assess normal distribution. Variables exhibiting a normal distribution were analyzed using the Student’s t-test or paired-sample t-test where applicable, while non-normally distributed variables were analyzed using the Mann-Whitney U test or Wilcoxon sign-rank test where applicable. The relationships between variables and ASMI change were analyzed using correlation coefficients and linear regression analyses. The ability of the variables to predict ASMI reduction was assessed using receiver operating characteristic (ROC) curve analysis. All analyses were performed using IBM SPSS Statistics 26.0, with statistical significance set at *p*-value < 0.05.
